# In Vitro Evaluation of Prebiotic Properties of a Commercial Artichoke Inflorescence Extract Revealed Bifidogenic Effects

**DOI:** 10.3390/nu12061552

**Published:** 2020-05-26

**Authors:** Pieter Van den Abbeele, Jonas Ghyselinck, Massimo Marzorati, Agusti Villar, Andrea Zangara, Carsten R. Smidt, Ester Risco

**Affiliations:** 1 ProDigest BV, Technologiepark 82, 9052 Ghent, Belgium; Pieter.VandenAbbeele@prodigest.eu (P.V.d.A.); Jonas.Ghyselinck@prodigest.eu (J.G.); massimo.marzorati@prodigest.eu (M.M.); 2 Center of Microbial Ecology and Technology, Ghent University, 9000 Ghent, Belgium; 3 Euromed S.A., C/Rec de Dalt, 21-23, Pol. Ind. Can Magarola, Mollet del Valles, 08100 Barcelona, Spain; avillar@euromed.es (A.V.); ERisco@euromed.es (E.R.); 4 Centre for Human Psychopharmacology, Swinburne University, Melbourne, VIC 3122, Australia; 5 Smidt Labs, LLC, Sandy, UT 84092, USA; csmidt@smidtlabs.com; 6 Unitat de Farmacologia i Farmacognòsia, Facultat de Farmàcia, Universitat de Barcelona, Av. Joan XXIII, s/n. E-08028 Barcelona, Spain

**Keywords:** bifidobacteria, colon, fermentation, microbiota, prebiotic, SHIME^®^, artichoke

## Abstract

Background: Prebiotics used as a dietary supplement, stimulate health-related gut microbiota (e.g., bifidobacteria, lactobacilli, etc.). This study evaluated potential prebiotic effects of an artichoke aqueous dry extract (AADE) using in vitro gut model based on the Simulator of Human Intestinal Microbial Ecosystem (SHIME^®^). Methods: Short-term colonic fermentations (48 h) of AADE, fructo-oligosaccharides (FOS), and a blank were performed. Microbial metabolites were assessed at 0, 6, 24, and 48 h of colonic incubation via measuring pH, gas pressure, lactate, ammonium, and short-chain fatty acids (SCFAs) levels. Community composition was assessed via targeted qPCRs. Results: After 24 and 48 h of incubation, bifidobacteria levels increased 25-fold with AADE (*p* < 0.05) and >100-fold with FOS (*p* < 0.05) compared to blank. *Lactobacillus* spp. levels only tended to increase with AADE, whereas they increased 10-fold with FOS. At 6 h, pH decreased with AADE and FOS and remained stable until 48 h; however, gas pressure increased significantly till the end of study. Acetate, propionate, and total SCFA production increased significantly with both at all time-points. Lactate levels initially increased but branched SCFA and ammonium levels remained low till 48 h. Conclusion: AADE displayed prebiotic potential by exerting bifidogenic effects that stimulated production of health-related microbial metabolites, which is potentially due to inulin in AADE.

## 1. Introduction

Human gut microbiota consist of over 35,000 bacterial strains, encompassing beneficial and pathogenic species; however, the predominance of positively affecting microbes ensure our well-being [1]. Human gut microbiota are dominated by two main phyla, Firmicutes (including *Lactobacillus* spp.) and Bacteroidetes that are susceptible to alterations. Other phyla are Actinobacteria (including *Bifidobacterium* spp.), Proteobacteria, Fusobacteria, and Verrucomicrobia. Spatial and temporal discrepancies in gut microbial distribution contribute toward specific metabolic, immunological, and gut-protective functions throughout an individual’s life span [2,3]. Characterization of such discrepancies can help identify gut-related abnormalities and play an important role in ensuring good health [4].

Prebiotics were first defined as, “Nondigestible food ingredients that beneficially affect a host by selectively stimulating growth and/or activity of one or a limited number of bacteria in the colon that are recognized to improve host health” [5]. Dysbiosis of microbial populations has been postulated as one of the reasons for metabolic disorders such as obesity, type 2 diabetes, and nonalcoholic fatty liver diseases. As prebiotics alter microbiota positively, their use as dietary supplements could effectively improve overall host health [6]. Fructo-oligosaccharides (FOS) are prebiotics that are plant-derived, naturally occurring oligosaccharides, indigestible by human enzymes, and can thus reach the colon unaltered [7]. Daily intake of FOS can increase bifidobacteria counts, a member of the indigenous gut microbiota. However, certain individuals are more sensitive to effects of FOS and suffer side effects such as itching in the throat; puffiness in the eyes, face, and mouth; dizziness; light headedness; fainting; gas; bloating; and itching of the skin [8,9].

There is a constant need for new prebiotics that can target specific bacterial species and most approaches have focused on non-digestible oligosaccharides, such as galacto-oligosaccharides, soya-oligosaccharides, isomaltooligosaccharides, gluco-oligosaccharides, xylo-oligosaccharides, lacto-sucrose, and inulin-type fructans. However, they are known to have varied prebiotic potential. Inulin has been demonstrated to positively alter gut microbiota in a dose range of 4 to 40 g/d [10,11,12,13,14,15,16]. Whole food sources, such as artichoke (*Cynara scolymus* L.), chicory (*Cichorium intybus*) roots, and garlic (*Allium sativum*) are rich in inulin and dietary fibers. Inulin from artichoke is recognized to have the highest degree of polymerization known in plants. Degree of polymerization directly contributes to prebiotic effects and persistence in the colon [17]. Inulin promotes host health by positively altering the bacterial metabolites mediated via stimulation of different metabolic pathways within the gut microbial community. Acetate, propionate, and butyrate are the most crucial metabolites. By acidifying the colonic environment, short-chain fatty acids (SCFA) promote growth of beneficial bacteria such as bifidobacteria and lactobacilli, which inhibit the growth of pathogenic bacteria [18]. Additionally, bifidobacteria and lactobacilli exert immunomodulatory activity that contributes to the host defense [19]. Prebiotic potential of artichoke has been demonstrated in several clinical studies and the effect was mainly mediated via increase in *Bifidobacterium* spp. in the gut [20,21]. In addition to inulin, artichoke contains polyphenols, such as dicaffeoylquinic acids and flavonoids, which provide additional nutritional values proposing a novel holistic approach to whole digestive health [22,23].

In the present study, we aimed to evaluate the prebiotic effects of artichoke aqueous dry extract (AADE) through an in vitro approach of highly controlled conditions using short-term colonic incubations based on the Simulator of Human Intestinal Microbial Ecosystem (SHIME^®^) model [18]. In vitro models offer certain advantages, first they allow dynamic monitoring of gut microbiome at the site of fermentation under a controlled environment and second, in vitro models help avoid large variability that arise during in vivo evaluations owing to host-derived factors such as amount of food intake, immune system, enzyme levels, or transit time. Lastly, using molecular detection methods, microbial changes can be evaluated in detail. Artichoke aqueous dry extract is a standardized herbal powder extract prepared from the edible part of artichoke (*Cynara scolymus* L.); cultivated in Spain, and extracted by the Pure-Hydro Process™ using only water (instead of organic solvents), AADE can be safely used in foods and food supplements.

## 2. Materials and Methods

### 2.1. Chemicals and Reagents

All chemicals were obtained from Sigma-Aldrich (Overijse, Belgium), unless stated otherwise. The test product AADE, also known as Cynamed™, was provided by Euromed S.A. (Mollet del Valles, Barcelona, Spain). It is derived from the edible part of the artichoke plant (*Cynara scolymus* L.) cultivated in Mediterranean regions of Spain. The AADE was prepared in accordance with the European Pharmacopoeia monograph extracts (Extracta) (No. 0765) using a proprietary water-based extraction process [24]. This process starts with the milling of dried Artichoke immature edible inflorescences that are extracted with ultrapure water at a temperature between 80 °C and 90 °C. The miscella of extract is filtered until transparency and concentrated under vacuum until a soft paste is obtained that is subsequently dried in a vacuum belt dryer and finally milled to a fine powder. The AADE used in the current study has an exact content of 9.1% caffeoylquinic acids expressed as chlorogenic acid by HPLC and an exact content of 32.2% inulin determined by HPLC. As a nutritional analysis of the AADE, the amount of total carbohydrates is 77% and the amount of protein 8.1% with a negligible content of fat. The FOS preparation used as a positive control in the current study had a purity of 89% FOS with 8% sugar residues. While the degree of polymerization of the ingredient varied between 2 and 10, it was on average 4.

### 2.2. Short-Term Colonic Fermentation

Short-term colonic fermentations were performed as described recently [18]. Briefly, colonic background medium containing 5.2 g/L K_2_HPO_4_, 16.3 g/L KH_2_PO_4_, 2.0 g/L NaHCO_3_ (Chem-lab NV, Zedelgem, Belgium), 2.0 g/L Yeast Extract, 2.0 g/L pepton (Oxoid, Aalst, Belgium), 1.0 g/L mucin (Carl Roth, Karlsruhe, Germany), 0.5 g/L L-cystein, and 2.0 mL/L Tween80 (Sigma-Aldrich, Bornem, Belgium) was added to incubation reactors (90 vol%), already containing the correct amount of the test products for obtaining a final concentration of 0 g/L (Blank) or 5 g/L (for both AADE and FOS), respectively. The reactors were sealed and anaerobiosis was obtained by flushing with N_2_. Subsequently, fresh fecal material of a healthy human donor (no history of antibiotic use in the six months preceding the study) was collected (according to the ethical approval of the University Hospital Ghent with reference number B670201836585; 06/08/2018). After preparation of an anaerobic fecal slurry, this was inoculated at 10 vol% in the aforementioned medium. All incubations were performed in biological triplicate for 48 h at 37 °C under anaerobic conditions with continuous shaking (90 rpm).

### 2.3. Microbial Metabolic Activity Analysis

Microbial metabolic analyses were performed on samples collected at 0, 6, 24, and 48 h of colonic incubation and levels of pH (Senseline F410; ProSense, Oosterhout, The Netherlands), gas pressure (hand-held pressure indicator CPH6200; Wika, Echt, The Netherlands), lactate, ammonium, and short-chain fatty acids (SCFAs) were measured. Acetate, propionate, butyrate, and branched CFAs (BCFAs) (isobutyrate, isovalerate, and isocaproate) were quantified as described by De Weirdt et al. [25] via GC-FID after performing a diethyl ether extraction. Lactate determination was performed using a commercially available enzymatic assay kit (R-Biopharm, Darmstadt, Germany) as per the manufacturer’s instructions. Ammonium analysis was performed using a KjelMaster K-375 device (Büchi, Hendrik-Ido-Ambacht, The Netherlands), wherein ammonium in the sample was liberated as ammonia by addition of 32% NaOH. The released ammonia was then distilled from the sample into a 2% boric acid solution and was titrimetrically determined with a 0.02 M HCl solution.

### 2.4. Microbial Community Analysis

At the start of colonic incubation and after 24 and 48 h, samples were collected for microbial community analysis. DNA was isolated using the protocol as described by Vilchez-Vargas et al. [26], starting from pelleted cells originating from 1 mL luminal sample. Subsequently, quantitative polymerase chain reaction (qPCR) assays for Bacteroidetes, Firmicutes, *Lactobacillus* spp. (Firmicutes phylum), *Bifidobacterium* spp. (Actinobacteria phylum), and *Akkermansia muciniphila* (Verrucomicrobia phylum) were performed using a StepOnePlus™ real-time PCR system (Applied Biosystems, Foster City, CA, USA). Each sample was analyzed in triplicate. Standard curves for all the different runs had efficiencies between 90–105%. All protocols were initiated for 10 min at 95 °C and terminated with a melting curve from 60 °C to 95 °C. Cycling programs included 40 cycles with a denaturation step of 15 s at 95 °C, an annealing step of 30 s at 60 °C, and an elongation step of 30 s at 72 °C in each cycle. Descriptions of primers used are presented in Table 1.

Besides presenting the absolute levels of the different groups, the ratio between the obtained levels at 24 h and 48 h versus 0 h were calculated for the blank, AADE, and FOS-treated microbiota.

### 2.5. Statistics

To evaluate differences in microbial metabolites and microbial community composition between blank and treatment incubations at the different time points, a two-way ANOVA with Tukey multiple comparisons test was performed. Differences were found significant if *p* < 0.05. Statistical analysis was performed with the GraphPad Prism software (version 8.3.0, San Diego, USA).

## 3. Results

### 3.1. Microbial Composition

**Figure 1:**
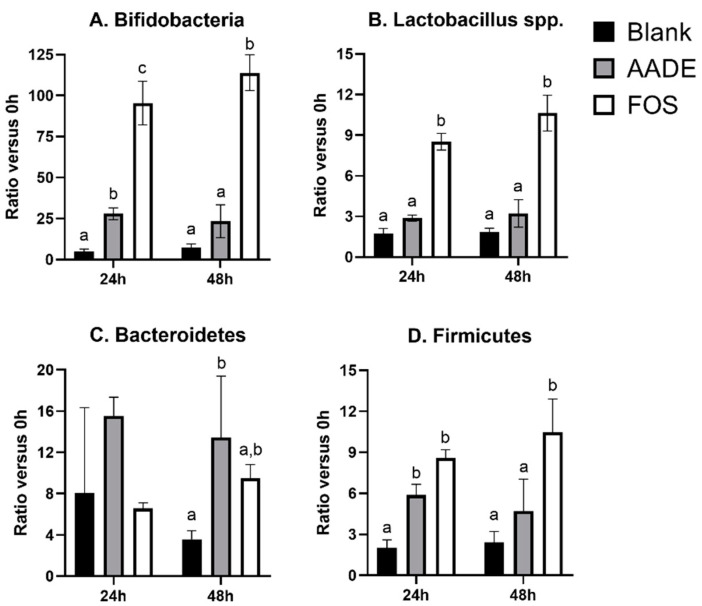
Mean (±standard deviation) ratios of (**A**) *Bifidobacterium* spp., (**B**) *Lactobacillus* spp., (**C**) Firmicutes, and (**D**) Bacteroidetes levels after 24 h or 48 h of treatment of a simulated colonic microbiota with 5 g/L AADE (artichoke aqueous dry extract) or FOS (fructo-oligosaccharides) versus the initial levels (24 h/0 h or 48 h/0 h, respectively) as measured via quantitative polymerase chain reaction (qPCR). For each microbial group and within each time point (24 or 48 h), a bar indicated with a different letter (a, b, or c) indicates a statistical difference between AADE, FOS, and/or the blank at a given time point, as tested with a two-way ANOVA with post-hoc Tukey test (*p* < 0.05). In contrast, when at least one letter is shared between two bars, there was no significant between these treatments.

While the absolute levels of each of the five targeted microbial groups (*Bifidobacterium* spp., *Lactobacillus* spp., Bacteroidetes, Firmicutes, and *Akkermansia muciniphila*) at each of the three time points (0/6/48 h) are presented in Table 2, the factor increase versus 0 h is presented in Figure 1 for the four microbial groups for which there were significant changes between the treatments (all except *Akkermansia muciniphila*). First, both at 24 h and 48 h, bifidobacteria levels were significantly increased versus the blank for AADE but especially for FOS (Table 2). This was reflected by ~25-fold and ~100-fold increased levels versus 0 h for AADE and FOS, respectively; both after 24 h and 48 h of incubation (Figure 1A). Additionally, *Lactobacillus* spp. were stimulated more profoundly for FOS with ~10-fold increased levels versus 0 h at 24 and 48 h (Figure 1B). AADE exerted more attenuated effects on *Lactobacillus* spp. levels with only statistically significantly increased absolute levels at 48 h. Incubation with FOS increased absolute Firmicutes levels at all time points, while for AADE the increase was only significant at 24 h (Table 2 and Figure 1C). Finally, AADE increased Bacteriodetes levels versus the blank at 48 h (Table 2 and Figure 1D), while FOS decreased *Akkermansia muciniphila* levels versus AADE after 48 h of incubation (Table 2).

### 3.2. pH

A more profound decrease in pH was observed with FOS and to a lesser extent also with AADE compared with blank at 6 h (*p* < 0.05). The pH continued to decrease until 24 h and remained stable thereafter, indicating continued microbial fermentation (Table 3).

### 3.3. Gas Pressure

As noted in Table 3, as compared with the blank, gas pressure was significant with AADE and FOS on all the time points along the incubation. On all time points, gas production was significantly higher for FOS versus AADE (Table 3).

### 3.4. Lactate and Carbohydrate (SCFAs, Acetate, Butyrate, and Propionate) and Protein Metabolites (BCFAs and Ammonium)

Compared with the blank, total SCFAs were significantly increased with AADE and FOS at all time-points of incubation (*p* < 0.05), which reflected enhanced microbial metabolic activity upon AADE/FOS administration. However, the overall increase in total SCFAs was higher with FOS compared with AADE (Table 4). Similar patterns of increase in acetate and propionate levels were observed with AADE and FOS as for the total SCFA (Table 4). In contrast, butyrate concentrations were only significantly increased for FOS, and this after 24 h and 48 h.

As shown in Table 4, no BCFAs were produced during the initial 6 h of incubation in AADE and FOS. After 24 h of incubation, there was a similar production of BCFAs in the blank (1.34 ± 0.24) and upon AADE treatment (1.70 ± 0.37). In contrast, no BCFAs were produced upon FOS administration at the 24 h time point. After 48 h of incubation, BCFAs were produced but were significantly (*p* < 0.05) lower for both AADE (3.42 ± 0.02) and especially FOS (0.34 ± 0.26) when compared with the blank (4.01 ± 0.13). The results for ammonium, another marker for protein fermentation, were similar to those for BCFAs, indicating reduced protein fermentation upon AADE and especially FOS administration. Lactate levels were high (*p* < 0.05) with AADE and even further increased for FOS compared with blank after the initial 6 h of incubation. Thereafter, lactate levels decreased indicating lactate consumption.

## 4. Discussion

In the present study, although the effects of AADE on microbial activity and composition were milder as compared to the “gold standard” prebiotic FOS, AADE demonstrated marked prebiotic potential. First, AADE significantly decreased pH and increased gas production, which indicated overall increased microbial activity upon administration of the test product. Saccharolytic metabolites such as acetate and propionate, and thus also total SCFAs, increased, while levels of proteolytic metabolites, BCFAs, and ammonium, significantly decreased upon AADE administration at 48 h. A key finding of this study was the growth-promoting action of AADE, mostly on bifidobacteria which are regarded as health-related members of the intestinal microbiome. Further, AADE also affected Bacteroidetes, Firmicutes, and *Lactobacillus* spp. levels.

Based on results of this study, bifidogenic effects of AADE were milder, yet in the same order of magnitude as those of FOS. These findings were similar to those of a previous in vitro study conducted by Barszcz M et al. [28]. Bifidogenic effects were also reported in healthy volunteers [20,21]. The bifidogenic effect of artichoke has been attributed to its inulin content. Inulin exerts most physiological changes through the bacterial metabolites. SCFAs are some of the important metabolites that acidify the colonic environment promoting growth of beneficial bacteria, such as *Lactobacillus* spp. and bifidobacteria, and prevent growth of pathogenic bacteria [30,31].

Moreover, in our study, lactate was produced during the initial 6 h of incubation. Subsequently, lactate was consumed and coincided with an increase in propionate levels for both AADE and FOS, with most marked stimulations being noted for FOS. Butyrate was not stimulated by AADE, suggesting that the majority of lactate (that can be used as a substrate for both propionate and butyrate), was cross-fed to propionate upon AADE supplementation. Some Negativicutes (family Veillonellaceae, phylum Firmicutes) are shown to form propionate [32] and could potentially explain the increase of Firmicutes that was observed for AADE after 24 h in our study. Bacteroidetes also contain potent propionate producers [33,34] and could have further contributed to propionate production upon AADE supplementation since AADE also stimulated this phylum in our study. These alterations in propionate levels correlate with the inulin content of the artichoke [18]. Propionate metabolites have been shown to reduce cholesterol and fatty acid synthesis in liver, improve glucose metabolism, and regulate immune status in adipose tissue, and thus elicit health-promoting activities [18,35]. Finally, another key propionate producer is the mucin-degrading, acetate and propionate producing, *Akkermansia muciniphila.* This taxon was not increased for either AADE or FOS and even decreased upon FOS administration. This was likely due to the fact that FOS more strongly decreased the pH (to 5.66 within 24 h), which is a pH at which *Akkermansia muciniphila* is unable to grow [36]. In vivo, such lower pH could however boost mucin secretion and result in enhanced mucin degradation by *Akkermansia muciniphila* in the distal colon, as shown for inulin in humanized rats [37].

Similarly, acetate and lactate can be cross-fed to butyrate by members of the Ruminococcaceae, Lachnospiraceae, Clostridiaceae, Eubacteriaceae, all members of the Firmicutes phylum [18,38]. Butyrate is a major energy source for the gut microbiota and may also reduce oxidative stress, improve gut function, and restrict inflammatory response. In this study, butyrate levels were increased majorly with FOS and were usually produced during the later stage of incubation period. As our study duration was limited to 48 h, additional studies with longer incubation periods are warranted to make accurate conclusions. Moreover, cross-feeding between microbial communities should be taken into account when drawing definite conclusions [39].

Furthermore, propionate and acetate have been shown to stimulate release of peptide hormones leading to short-term signaling of satiation and satiety to appetite centers in the brain, resulting in reduced food intake by the host [35,40,41]. Several metabolic disorders such as obesity, insulin resistance, and metabolic syndrome are associated with impaired carbohydrate and lipid metabolism by the host, and are accompanied by changes in the gut microbiota [32]. Inulin could stimulate different metabolic pathways within the gut microbial community and could potentially elicit varied health-promoting activities [18].

Ammonia and BCFAs are toxic metabolites produced from protein fermentation [39]. In this investigation, the reduction in BCFAs and ammonia production in part explains the increase in carbohydrate metabolism. In vivo studies have also demonstrated that generation and accumulation of ammonia can be reduced by lowering protein supply and by colonic fermentation of suitable non-digestible carbohydrates from food [39,42].

Short-term colonic incubations have often been used to gather information on the prebiotic potential of novel ingredients. Results of the present study using an incubation strategy based on the SHIME^®^ model indicate the prebiotic potential of AADE. These findings could be further validated using different models, such as M-SHIME^®^ (Mucosal Simulator of the Human Intestinal Microbial Ecosystem) which focuses not only on luminal but also mucosal gut-colonizing microbes [43]. Moreover, studies with repeated administration are required in order to simulate gradual changes that occur in vivo with long-term use and to assess any beneficial microbial shift.

## 5. Conclusions

The present preliminary evaluation, conducted using the SHIME^®^ model, demonstrated that AADE has promising prebiotic potential. Incubation with AADE resulted in an increase of beneficial microbes, which was correlated with their metabolite profile. The promising results of this study justify future investigations using multiple doses in upgraded models to further validate these findings.

## Figures and Tables

**Table 1:** Primers used for quantitative polymerase chain reaction (qPCR) quantification of species-specific 16S rDNA.

Target Species	Primer Sequences 5′-3′ and 3′-5′
Bacteroidetes [27]	GGAACATGTGGTTTAATTCGATGAT
	AGCTGACGACAACCATGCAG
Firmicutes [27]	GGAGCATGTGGTTTAATTCGAAGCA
	AGCTGACGACAACCATGCAC
*Lactobacillus* spp. [20]	AGCAGTAGGGAATCTTCCA
	CGCCACTGGTGTTCYTCCATATA
*Bifidobacterium* spp. [28]	TCGCGTCYGGTGTGAAAG
	CCACATCCAGCYTCCAC
*Akkermansia muciniphila* [29]	CAGCACGTGAAGGTGGGGAC
	CCTTGCGGTTGGCTTCAGAT

**Table 2:** Mean (±standard deviation) levels of microbial groups as measured via quantitative polymerase chain reaction (qPCR) after 0, 24, and 48 h of treatment of a simulated colonic microbiota with 5 g/L AADE (artichoke aqueous dry extract) or FOS (fructo-oligosaccharides). For each microbial group and within each time point (24 h or 48 h), a value indicated with a different letter (a, b, or c) indicates a statistical difference between AADE, FOS, and/or the blank, as tested with a two-way ANOVA with post-hoc Tukey test (*p* < 0.05). In contrast, when at least one letter is shared between two treatments, there was no significant between these groups.

	Levels of Microbial Groups (log (16S rRNA Copies/mL))						
Incubation Time (h)	0 h	24 h			48 h		
		Blank	AADE	FOS	Blank	AADE	FOS
Firmicutes	9.96 ± 0.36	10.36 ± 0.13 ^a^	10.83 ± 0.06 ^b^	11.00 ± 0.03 ^b^	10.44 ± 0.14 ^a^	10.70 ± 0.25 ^a^	11.08 ± 0.10 ^b^
Bacteroidetes	9.73 ± 0.46	10.64 ± 0.47	11.09 ± 0.05	10.72 ± 0.04	10.44 ± 0.10 ^a^	10.99 ± 0.23 ^b^	10.87 ± 0.06 ^a,b^
Bifidobacteria	8.26 ± 0.25	8.94 ± 0.13 ^a^	9.71 ± 0.06 ^b^	10.24 ± 0.06 ^c^	9.12 ± 0.14 ^a^	9.60 ± 0.22 ^b^	10.32 ± 0.04 ^c^
*Lactobacillus* spp.	6.58 ± 0.19	6.84 ± 0.10 ^a^	7.06 ± 0.03 ^a^	7.54 ± 0.03 ^b^	6.87 ± 0.06 ^a^	7.01 ± 0.15 ^b^	7.63 ± 0.06 ^c^
*Akkermansia mucinphila*	7.02 ± 0.51	8.23 ± 0.39	8.30 ± 0.03	7.72 ± 0.08	8.08 ± 0.17 ^a,b^	8.19 ± 0.25 ^b^	7.85 ± 0.08 ^a^

**Table 3:** Mean (±standard deviation) pH and gas pressure after 0, 6, 24, and 48 h of treatment of a simulated colonic microbiota with 5 g/L AADE (artichoke aqueous dry extract) or FOS (fructo-oligosaccharides). For each time point (0, 6, 24, or 48 h), a value indicated with a different letter (a, b, or c) indicates a statistical difference between AADE, FOS, and/or the blank as tested with a two-way ANOVA with post-hoc Tukey test (*p* < 0.05).

**Incubation Time (h)**	**pH**		
	Blank	AADE	FOS
0	6.49 ± 0.02	6.51 ± 0.00	6.51 ± 0.00
6	6.39 ± 0.01 ^a^	6.22 ± 0.01 ^b^	5.64 ± 0.13 ^c^
24	6.46 ± 0.02 ^a^	6.21 ± 0.01 ^b^	5.66 ± 0.02 ^c^
48	6.40 ± 0.04 ^a^	6.20 ± 0.02 ^b^	5.69 ± 0.03 ^c^
**Incubation Time (h)**	**Gas Pressure** **(kPa)**		
	Blank	AADE	FOS
6 h	13.2 ± 0.2 ^a^	22.8 ± 1.6 ^b^	29.9 ± 1.6 ^c^
24 h	27.3 ± 0.6 ^a^	45.5 ± 0.5 ^b^	52.0 ± 2.8 ^c^
48 h	31.4 ± 0.6 ^a^	48.8 ± 0.6 ^b^	54.2 ± 1.1 ^c^

**Table 4:** Mean (±standard deviation) carbohydrate- and protein-derived metabolites after 0, 24, and 48 h of treatment of a simulated colonic microbiota with 5 g/L AADE (artichoke aqueous dry extract) or FOS (fructo-oligosaccharides). For each endpoint and within each time point (24 h or 48 h), a value indicated with a different letter (a, b, or c) indicates a statistical difference between AADE, FOS and/or the blank, as tested with a two-way ANOVA with post-hoc Tukey test (*p* < 0.05). In contrast, when at least one letter is shared between two treatments, there was no significant between these groups.

Incubation Time (h)	6 h			24 h			48 h		
	Blank	AADE	FOS	Blank	AADE	FOS	Blank	AADE	FOS
Carbohydrate Metabolite Levels (mean ± SD) (mM)									
Acetate	8.1 ± 0.3 ^a^	17.2 ± 1.0 ^b^	31.4 ± 3.0 ^c^	19.2 ± 0.3 ^a^	34.9 ± 0.3 ^b^	40.1 ± 0.4 ^c^	20.8 ± 0.3 ^a^	37.1 ± 0.9 ^b^	43.7 ± 0.7 ^c^
Butyrate	0.41 ± 0.03	0.26 ± 0.05	0.48 ± 0.01	2.47 ± 0.03 ^a^	2.89 ± 0.53 ^a^	5.53 ± 0.64 ^b^	3.78 ± 0.02 ^a^	3.9 ± 0.55 ^a^	7.17 ± 0.22 ^b^
Propionate	3.5 ± 0.2 ^a^	7.6 ± 0.6 ^b^	8.7 ± 0.8 ^c^	7.1 ± 0.1 ^a^	15.6 ± 0.2 ^b^	23.6 ± 0.6 ^c^	7.8 ± 0.20 ^a^	16.3 ± 0.4 ^b^	24.1 ± 0.6 ^c^
Total SCFAs	12.1 ± 0.5 ^a^	25.0 ± 1.6 ^b^	40.6 ± 3.9 ^c^	31.6 ± 0.4 ^a^	56.7 ± 0.5 ^b^	69.5 ± 0.9 ^c^	38.3 ± 0.7 ^a^	65.6 ± 1.2 ^b^	75.7 ± 0.8 ^c^
Lactate (mean ± SD) (mM)	1.55 ± 0.03 ^a^	3.79 ± 0.04 ^b^	8.84 ± 1.16 ^c^	0.25 ± 0.01	0.33 ± 0.07	0.85 ± 0.78	0.49 ± 0.05	0.47 ± 0.21	0.10 ± 0.07
Protein Metabolite Levels (mean ± SD) (mg/L)									
BCFAs	0.10 ± 0.00	0.00 ± 0.00	0.00 ± 0.00	1.34 ± 0.24 ^a^	1.70 ± 0.37 ^a^	0.00 ± 0.00 ^b^	4.01 ± 0.13 ^a^	3.42 ± 0.02 ^b^	0.34 ± 0.26 ^c^
Ammonium	0 ± 0	0 ± 0	0 ± 0	328 ± 1 ^a^	322 ± 7 ^a^	122 ± 5 ^b^	414 ± 3 ^a^	384 ± 13 ^b^	1874 ± 12 ^c^

BCFAs, branched short-chain fatty acid; SCFAs, short-chain fatty acids.
